# Dermal Denticles of Three Slowly Swimming Shark Species: Microscopy and Flow Visualization

**DOI:** 10.3390/biomimetics4020038

**Published:** 2019-05-24

**Authors:** Katrine Feld, Anne Noer Kolborg, Camilla Marie Nyborg, Mirko Salewski, John Fleng Steffensen, Kirstine Berg-Sørensen

**Affiliations:** 1 Department of Physics, Technical University of Denmark, DK-2800 Kongens Lyngby, Denmark; katrine@feld.dk (K.F.); anne.kolborg@nbi.ku.dk (A.N.K.); CamillaMarieNyborg@hotmail.com (C.M.N.); msal@fysik.dtu.dk (M.S.); 2 Marine Biological Section, Department of Biology, University of Copenhagen, DK-3000 Helsingør, Denmark; jfsteffensen@bio.ku.dk

**Keywords:** shark skin, micro-PIV, microfluidics

## Abstract

Shark skin has for many years inspired engineers to produce biomimetic structures reducing surface drag or acting as an anti-fouling layer. Both effects are presumed to be consequences of the structure of shark skin that is composed of arrays of so-called dermal denticles. However, the understanding of the full functional role of the dermal denticles is still a topic of research. We report optical microscopy and scanning electron microscopy of dermal denticles from three slowly swimming shark species for which the functional role of the dermal denticles is suggested as one of defense (possibly understood as anti-fouling) and/or abrasion strength. The three species are Greenland shark (*Somnosius microcephalus*), small-spotted catshark (*Scyliorhinus canicula*) and spiny dogfish (*Squalus acanthias*). Samples were taken at over 30 different positions on the bodies of the sharks. In addition, we demonstrate that the flow pattern near natural shark skin can be measured by micro-PIV (particle image velocimetry). The microfluidic experiments are complemented by numerical flow simulations. Both visualize unsteady flow, small eddies, and recirculation bubbles behind the natural dermal denticles.

## 1. Introduction

The biology of elasmobranchs, including sharks, have intrigued marine biologists and paleontologists for several hundred years [1,2]. One of the intriguing points is the appearance of so-called placoid scales, or dermal denticles, in the skin of the sharks. Since the pioneering work of Wolf-Ernst Reif initiated in the 1970s, summarized e.g., in [3,4], the putative drag-reducing effects of these dermal denticles of the fastest-swimming sharks such as *Lamniformes* have triggered engineers to produce surfaces with structural elements that although enlarged in scale, were inspired by the shapes of these dermal denticles [5,6]. This putative drag-reducing function is seen in the fastest-swimming sharks and is supposedly optimized for their maximum swimming speed, obtained during bursting motion [3]. It is probably the most sought-after function in biomimetic engineering work inspired by shark skin. The putative drag-reducing effect of some of these engineered structures has, however, been shown to be limited [7] and for some conditions even drag increasing [7]. In recent studies, surfaces that were replicas of the natural structures were investigated [8,9], and discussions of the design include the importance of the flexibility of the substrate in which the dermal denticles reside [8,10] and the ability of the dermal denticles to bristle [11,12,13,14]. Also, the spacing and modifications of the patterning of the denticle-like structures are a topic of investigations related to the drag-reducing properties of the structured surface [10,15,16].

According to the original investigations of dermal denticles and resulting conjectures by Reif [3,17], the structure and role of dermal denticles is, however, richer than that of a drag-reducing surface. The paleontological investigations of Reif point to division of sharks into four groups, according to the putative function of their dermal denticles, as summarized in Figure 18 in [3]. In addition to drag reduction, dermal denticles of other groups of sharks are thus suggested to provide abrasion strength, defense, and luminescence. The latter is actually not achieved by the dermal denticles themselves but rather by photophores in other parts of the skin of the shark, and recent literature [18] suggests excluding the luminescent function and thus reduce the number of groups to three.

Dermal denticles believed to provide abrasion strength are postulated mainly for demersal species of sharks living near rocky surfaces whereas both abrasion strength and defense against ectoparasites are suggested for demersal species living in sandy or muddy areas [3]. Finally, the dermal denticles of slowly moving sharks living in open water are suggested to provide defense against ectoparasites [3]. It is unclear how defense should be understood in this context, and one may speculate of a relation to anti-fouling. Anti-fouling properties of direct replicas of dermal denticles from slowly moving sharks have been investigated [19], but the dermal denticle structures of fast-moving sharks have also inspired the creation of bioengineered surfaces that have been demonstrated to reduce fouling of small bacteria [20]. The structures of dermal denticles of sharks add to other natural structures in the quest for microstructured surfaces that would reduce fouling [20,21,22,23]. Naturally, the anti-fouling properties depend on flow conditions and the full understanding of the phenomenon is as yet unclear [19]. Thus, it appears that there is more to the story than the original hypotheses put forward by Reif while grouping species of sharks into four [3].

Our conjecture is that the nature of the flow pattern at length scales ranging from well below the size of the dermal denticle to several times the denticle size plays a role for the function of the dermal denticles. Thus, visualization of the flow surrounding the dermal denticles is relevant and interesting, and to perform this visualization, the method of micro-PIV is applied, to complement investigations in the literature visualizing the flow at larger scale using otherwise comparable technology [7,14,24,25]. In Particle Image Velocimetry (PIV), flow velocity fields are measured by imaging tracer particles in two consecutive photos taken in a short time. Velocities can be measured based on the correlation extracted from the image pair [26]. Micro-PIV is the microfluidic version [27] using microspheres and imaging through microscope optics. More elaborate techniques like stereoscopic micro-PIV or tomographic micro-PIV have been developed, see e.g., [28], but for these first reported micro-PIV measurements near natural shark skin samples, we apply the originally developed micro-PIV technology as commercially available, and discussed further in Section 5.

The exact size and distribution of dermal denticles depend on the actual position on the body of the shark. Therefore samples from sharks at selected positions on the body of the shark were investigated in detail, following an extension of the map in [3], similar to that applied by other authors [12,13], see Supplementary Figure S1. The detailed visualization of the structures of dermal denticles in the skin of the shark samples, and of their distribution and areal density, is conducted by use of scanning electron microscopy (SEM) and optical microscopy.

## 2. Results of Microscopy of Dermal Denticles

**Figure 1:**
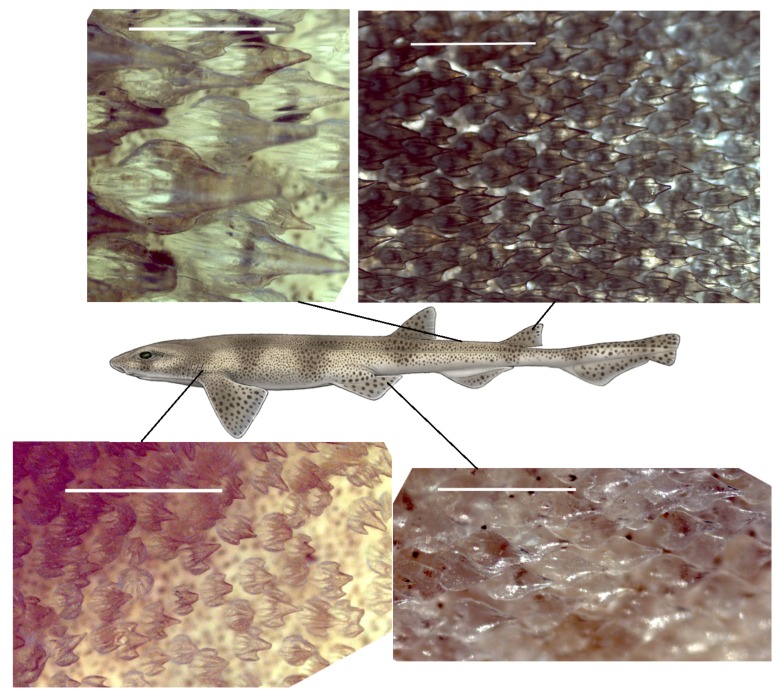
(Color online) Microscope images of denticles of the small-spotted catshark (male, TL 72 cm). All scalebars are 1 mm. Top left image with the longest measured denticles from the back of the body, area *b10* in Figure S1. The top right image shows smaller ribbed denticles from the second dorsal fin (area here denoted *d4*). A more random packing and direction of denticles is seen in the area behind the gills (*g2*), as indicated by the image in the bottom left. The last image shows denticles from the tip of the pelvic fin (area denoted *p8*) which are very smooth, almost drop-shaped, and transparent. The sketch in the middle is copyright Marc Dando and reprinted with his permission.

For the small-spotted catshark, detailed images of the dermal denticles were obtained by SEM with examples shown in supplementary information, Figure S2, and less detailed by optical microscopy. Indicative variations over the body are apparent from Figure 1, showing the dermal denticles visualized by optical microscopy. Sizes and areal densities of the dermal denticles were based on the optical microscopy images. From the microscope images of the denticles, the length of a few denticles and the total number of denticles in each frame were noted. Our investigations indicated variations in the crown length of the dermal denticles between 300 μm and 1 mm, with areal densities between 400 cm${}^{-2}$ and 2000 cm${}^{-2}$. As one could expect, the longer the denticles, the lower their areal density. However, the relation between the length and the density is not described well by any power law, for this species. Smaller dermal denticles are seen on the fins and near gills whereas larger dermal denticles are found on the body (see supplementary information, Figure S6).

In addition to SEM and optical microscopy to determine density and size of the dermal denticles, we investigated one alive, adult shark, in an attempt to decide if bristling of the dermal denticles may take place; bristling has been investigated in recent literature and may be important for the hydrodynamic properties of the skin [11,12]. The shark was briefly positioned with its fin or tail under a stereo microscope and bending was induced to search for signs of bristling. We did not see evidence for this type of repositioning of the dermal denticles (see supplementary information). More detailed investigations as in [11] were not possible with an alive specimen.

**Figure 2:**
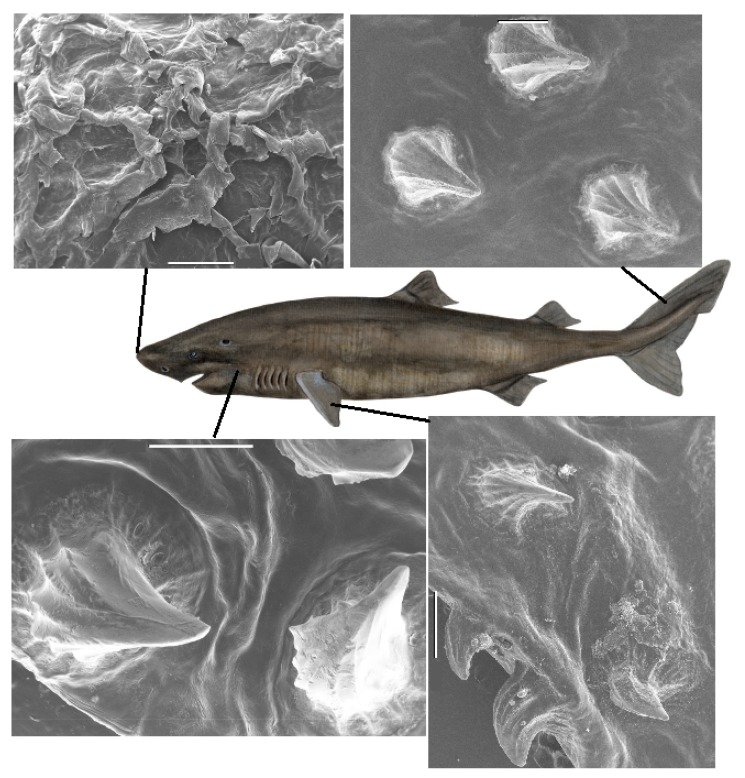
(Color online) SEM images of denticles of the Greenland shark (female, TL 470 cm) from positions *n1*, *b3*, *c2* and *p6* in Figure S1. Scalebars are 1 mm (*n1*, *b3*, *p6*) and 500 μm (*c2*). In area *n1*, the nose of the Greenland shark, a very different denticle structure than on the rest of the body is observed. On the top right of this image of area *n1*, a denticle that appears worn down is seen. In the area *b3*, it appears as if the directions of the tip of the denticles is somewhat random, whereas in other parts of the body, the denticles tend to point into the same direction, towards the tail. The central sketch of the shark is copyright Kirsten Hjørne and reprinted with her permission.

Also, for the Greenland shark, detailed images of the dermal denticles were obtained by SEM. Variations over the body are illustrated in Figure 2, with additional examples in supplementary information, Figure S3. The dermal denticles of the Greenland shark are particularly large, and on the microscopic scale, appear more sparsely distributed than those of the other shark species.

To determine the sizes and densities of the dermal denticles for determination of areal density and average size, skin samples were visualized with optical microscopy, with additional sample images available in the supplementary information (Figures S4 and S5). The logarithm of the density appears roughly inversely proportional to the length of the denticles. Also, for the Greenland shark, smaller dermal denticles are found on the fins and near the gills and larger denticles on the body (see supplementary information, Figure S6). Based on the shape of these dermal denticles, bristling is in our view unlikely.

**Figure 3:**
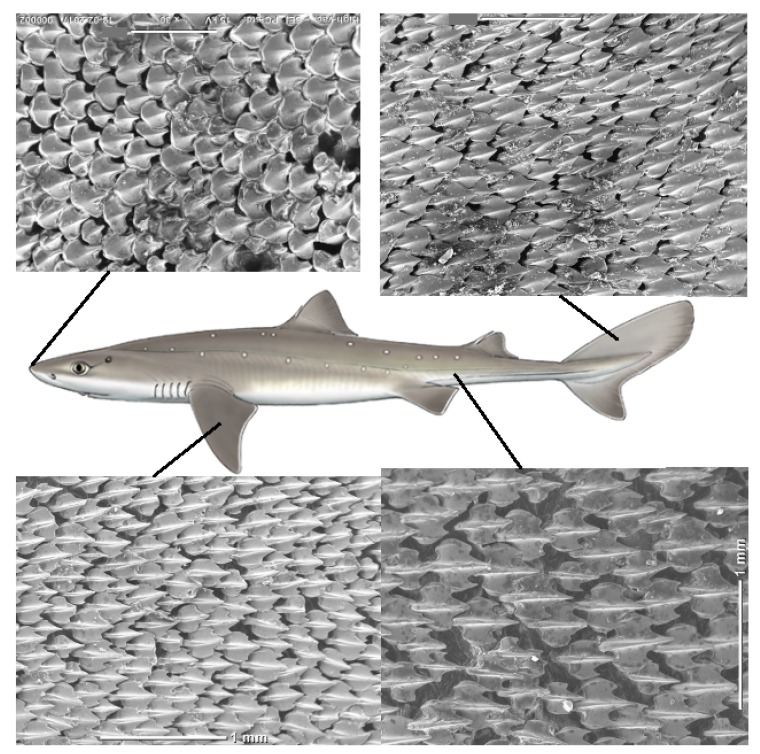
(Color online) Scheme showing the dermal denticles in different areas of the spiny dogfish (female, TL 76 cm). The SEM pictures are from top left to bottom right respectively showing dermal denticles from; the nose (*n1*), the caudal fin (*c1*), the pectoral fin (*p2*) and at the back of the body (*b11*). All scale bars are 1 mm. The central illustration of the shark is copyright Marc Dando and reprinted with his permission.

The dermal denticles of the spiny dogfish were comparably smaller and all images were obtained by SEM, with examples shown in Figure 3. Also, for this species, the larger the denticle, the lower the areal density (see supplementary information, Figure S7). The possibility for bristling was not investigated as samples had dried in the freezer before our experiments were conducted.

## 3. Results of Flow Measurements

The previous section described our microscopic investigation of many samples of denticles from several positions on the shark. In this section, we select a few of these to demonstrate microscopic flow visualization near real shark skin by micro-PIV, under microflow conditions for the first time.

### 3.1. Selected Results from Each of the Three Shark Species

In our micro-PIV experiments with samples from the small-spotted catshark, the flow was kept at 5 mL/min throughout the whole experiment through the chamber with a cross-section of 3 mm × 4 mm, corresponding to a flow velocity of approximately 7 mm/s along the chamber. With a typical length of 600μm of the dermal denticle as the length scale, this flow velocity corresponds to a Reynolds number of 4. Fluorescent seeding particles with a diameter of 0.86 μm were added to the water used in the flow experiment. Measurements taken from a side view with a 10× objective provided an image of a whole denticle. The calculated PIV vector fields did not show any flow close to the denticles (data not shown). Measurements with the 20× objective were conducted both near to and further from the dermal denticle. In all of these, 30 picture pairs were taken with a time delay of 8 ms, 10 ms and 12 ms between the paired images. A vector field is calculated for each of the 30 picture pairs using standard PIV processing tools, thus creating a short movie of 30 frames. The relatively low number of picture pairs was a compromise that ensured constancy in the overall imposed flow while still obtaining trustworthy micro-PIV vector fields. A time-averaged vector field of the 30 frames was then calculated. At this relatively low flow speed, the streamlines point almost straight away from the skin but vortices appear under the tips of the denticles (see supplementary information, Figure S8).

**Figure 4:**
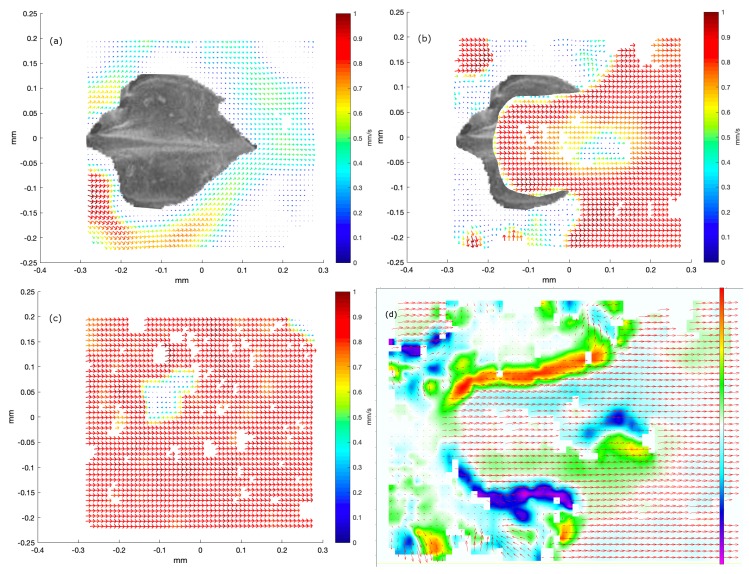
Results of micro-PIV measurements in three layers above a dermal denticle in the *b1* area of the dogfish (female, TL 99 cm). In all images, the size of the area visualized is 580 μm in width and 450 μm in height. To illustrate the position of the denticle, a single denticle is extracted from an SEM image from the *b1* area, scaled and overlaid with the image extracted from the PIV processing software. Positions of neighboring denticles correspond to white areas with no flow field. Part (**a**) illustrates both the structure of the denticle as seen from above and the flow around it, very close to the skin. The local flowspeed is indicated both by the length of the vector and by the color, with the colormap indicated to the right. Part (**b**) shows the velocity field just above the dermal denticle, in this plane vorticity may be found in the rectangle x∈[−0.05,0.1] and y∈[0.1,0.2]. The dermal denticle is cropped to only be overlaid in a region in which flow was not observed. Another signature of vortex formation appears in the lower left corner, rectangle with x∈[−0.3,−0.1] and y∈[−0.22,−0.15]. As in part (**a**), the local flow speed is indicated both by the length of the vector and by the color. Part (**c**) shows the flow well above the dermal denticle, now very uniform in the overall flow direction. The local flow speed is indicated both by the length of the vector and by the color. Part (**d**) shows a map of the rotation field, for the velocity field also shown in part (**b**). The color map indicates the rotation in units of 1/s, spanning the range [−450 s${}^{-1}$, 450 s${}^{-1}$].

For the spiny dogfish, results visualizing the flow above a denticle are illustrated in Figure 4. Because of the relatively small size of the denticles from the spiny dogfish, it is possible to get an entire denticle along with the surrounding flow in focus on one image, using the 10× objective. The denticle is seen from above, in a configuration with the overall direction of the flow from left to right, as in all previous figures. As a denticle is put in focus, most of the image is occupied by the tip of the denticle and the flow behind. This captures vortex formation just behind the denticle. For reference, an SEM picture of a denticle from the same area, in focus, is overlaid with the image of the flow field. Here, the three ridges, from where the vortex behavior of the fluid is expected to appear, are clearly observed. Vector fields in three planes above the denticle or including it are shown. Part (a) of the figure illustrates a plane where the denticle is clearly in focus. As one can observe, the focus plane is too close to the denticle, which implies that no seeding particles, or only seeding particles with no velocity, are tracked across the denticle. However, a very uniform flow is formed around the denticle. As the focus plane moves further away from the denticle, strong shear appears at the three ridges of the denticle. According to the small displacement of the focus plane, the extent of the vorticity is limited, which makes it difficult to locate the exact layer of vorticity. As soon as another focus layer than the one with the shearing flow lines is chosen, the flow becomes fairly uniform and undisturbed again. Some experiments show evidence of a very disturbed flow at the layer closest to the focus layer with the appearance of circulation. This does to a varying degree visualize that the vorticity extends through more fluid layers, or, at least, causes disturbances in the nearby layers. Yet, in the layer shown in part (b) of the figure, rotation in the flow is observed at the outer ridges of the denticle, along with some disturbance in the flow at the center ridge. The lack of a well-defined vorticity layer at the center ridge leads to the assumption that the tip is positioned at a different distance from the skin than the remaining ridges. Therefore, the vorticity could be appearing in another focus layer. The ambient zero velocity vectors in the left side of the image, where we have overlaid part of the same SEM picture as in part (a), demonstrate that the focus layer is still close enough to the surface of the denticle, for some of the seeding particles to be slowed down completely. Hence, the vorticity structures appear right above the surface. The rotation field (along the perpendicular axis), obtained in the same focus layer, is shown in part (d) of the figure.

**Figure 5:**
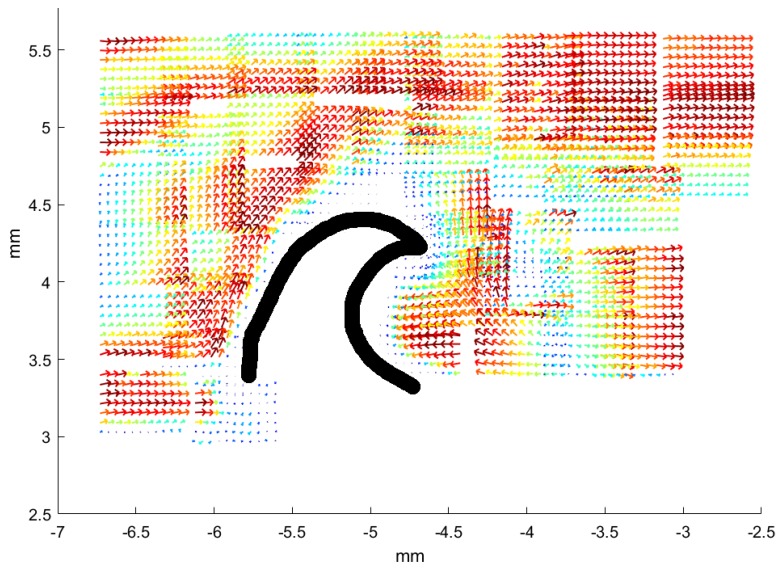
Average vector field of the flow near denticles from the Greenland shark, sample from position *b12*. The image is obtained by patching 48 individual images from fields-of-view obtained with the 10× objective and the PIV processing software. The denticle is “visible” as the white area in the middle with no flow pattern; to guide the eye a black handdrawn line sketches the outline of the denticle. Each of the 48 individual images cover an area of width 580 μm, and height 450 μm. Within each of the 48 individual images, the colormap is similar to that in Figure 4 such that the longest velocity vector is very dark red and the shortest are dark blue. Signatures of recirculation appears in the rectangle with x∈[−5,−4] and y∈[3.4,4.2].

**Figure 6:**
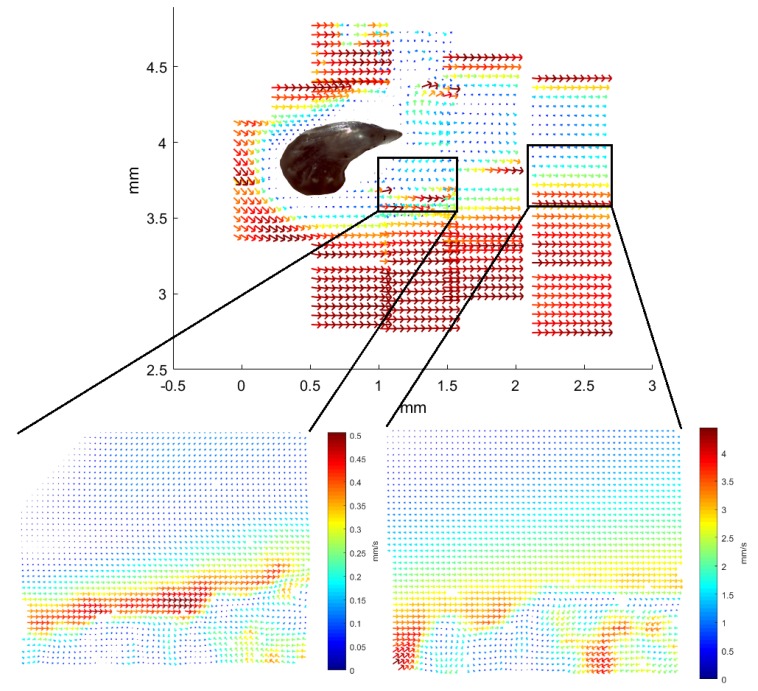
Average vector field of flow near denticles from the Greenland shark, sample from position *b12*, now seen from above. The image is patched from 21 individual images with fields-of-view obtained with the 10× objective and the PIV processing software. A microscope image of a denticle from the *b12* area is scaled and overlaid the patched image of the flow field to roughly show the position of the denticle, in the flow field it was “visible” as a white area in the middle with no flow pattern. Each of these 21 individual images cover an area of width 580 μm, and height 450 μm. The two insets or zooms are examples of such individual images and they illustrate details not visible in the combined image. Within each of the 21 individual images, the colormap is similar to that in Figure 4 such that the longest velocity vector is very dark red and the shortest are dark blue.

Time-averaged vector fields for the Greenland shark are shown in Figure 5 and Figure 6. Here, the 10× objective and 2 μm fluorescent microspheres were used. The tip of the denticle points to the right in both images, and the flow direction is also in that direction. Both images are patched from smaller images observing neighboring regions of the flow. The borders of the patched images are visible due to apparent jumps in the flow velocities. These apparent jumps occur as the small images are not acquired simultaneously. The flow conditions change slightly over time, so that neighboring measurements can differ more than would be expected for a fully static flow field. The time-averaged images were obtained from 30 instantaneous images, the highest number we could achieve that would still allow visualization of the flow in a sufficient number of regions to allow for visualization of the flow around an entire denticle. The small images in Figure 5 reveal that the flow is complex. The flow over the top of the denticles is in the mean flow direction. Behind the denticles, we find a recirculation bubble. The shear layers surrounding the recirculating flow are unstable so that the flow moves in an apparently random manner. From the large, patched image in Figure 5 it is, however, clear how the fluid moves over the top of the denticle. In the recirculation bubble behind the denticle, the flow appears to frequently change its direction. The measurements closest to the denticle were taken first while the measurements further to the right were obtained later.

To view the flow from above a denticle, a piece of skin is placed in a microfluidic chamber of horizontal configuration (see Figure 9c). Figure 6 shows the visualization of the flow in a plane parallel to the skin. The denticle is again located in the dark spot in the left part of the picture. With the fluid entering from the left side of the illustration, the interesting part is happening in the right of the picture. The patched 21 vector images present an overall consistent visualization of the flow. Only a single image provides a vector field different from the surrounding ones, in the top middle, with barely any velocity at all. Two vector fields are enlarged with the original resolution of the vector plot shown. In these original vector-field images, small eddies are seen in the bottom of the frames which are not present in the average image due to the loss of spatial resolution from the averaging over five by six velocity vectors.

### 3.2. Results for the Numerical Simulations of Flow Near Dermal Denticles

Numerical simulations of the flow in the vicinity of models of dermal denticles were conducted as described in Section 6. The dermal denticles in the numerical model are inspired by the shape of dermal denticles of the small spotted catshark, Figure S2.

**Figure 7:**
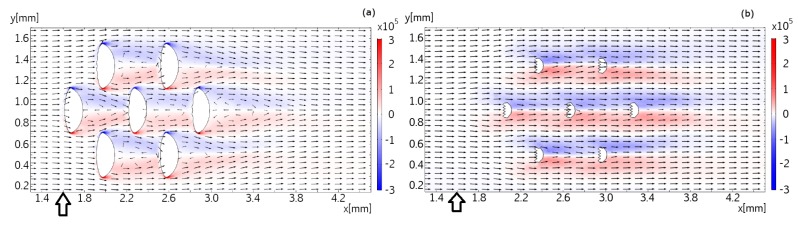
Flow pattern in the (x,y) plane, overlaid with *z* components of the vorticity field. No-slip boundary conditions are assumed at top ((x,y)-plane at z=0.5mm), bottom ((x,y)-plane at z=0mm) and sides ((x,z)-planes at y=0mm and y=4mm), as well as at the surface of the dermal denticles. Part (**a**) shows the flow field in a height of 100 μm from the bottom surface, part (**b**) in a height of 200 μm from the bottom surface. The vector field is plotted logarithmically with a range quotient of 1000 between shortest and longest arrow. The small marker below the ordinate axis is at position x=1.6μm in both cases.

**Figure 8:**
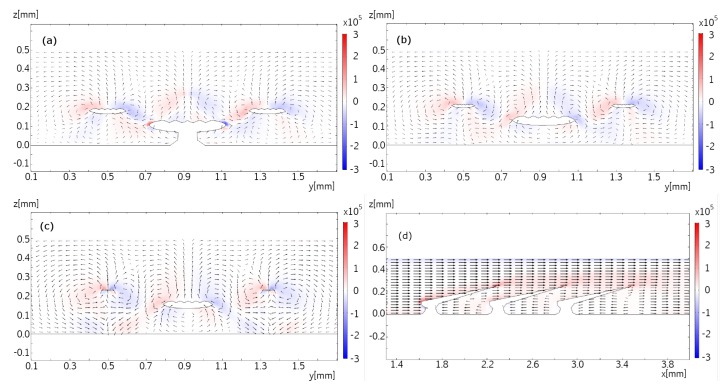
Parts (**a**–**c**) shows the flow pattern in the (y,z) plane, overlaid with *x* components of the vorticity field, with the same no-slip boundary conditions as in Figure 7. Panel (**a**) shows the flow in a reference position, and panels (**b**), and (**c**) are translated along *x* by 100 μm and 200 μm, respectively. Part (**d**) shows the flow pattern in the (x,z) plane, at the symmetry plane of the denticle, overlaid with *y* components of the vorticity field, with the same no-slip boundary conditions as in Figure 7. The vector field is plotted logarithmically with a range quotient of 1000 between shortest and longest arrow.

Examples of the resulting flow patterns in three orthogonal planes in (xy)-, (xz)-, and (yz)-directions are calculated with indicative examples shown in Figure 7 and Figure 8. The figures show the in-plane velocity components as vector arrows in addition to the vorticity component orthogonal to the plane as colors. The length of the arrows shows the logarithm of the in-plane velocity magnitude such that the flow velocity for the longest arrow is 1000 times larger than the flow velocity for the shortest arrow.

Figure 7 presents the flow in the (xy)-plane at distances of 100 μm and 200 μm above the wall. The dermal denticles extend up to about 300 μm above the wall, so that they form obstacles to the flow in these planes. At a distance of 100 μm to the wall, the recirculation bubble formed behind the dermal denticle extends to the next dermal denticle downstream. The velocity of the flow approaching each dermal denticle is therefore lower than it would be under free-stream conditions without neighboring dermal denticles upstream. The recirculation bubble is shorter at a distance of 150 μm from the wall (data not shown), and no recirculation bubble is found at a distance of 200 μm from the wall. The vorticity in the shear layers trailing off the dermal denticles is slightly higher than found in the micro-PIV measurements. The difference could be explained by the somewhat different geometry in the experiment compared to the simulation model.

Lastly, parts (a)–(c) of Figure 8 shows the flow in the cross-sectional planes (yz) catching an entire period of the repetitive pattern coming from the sequence of dermal denticles in flow direction. Each dermal denticle forms a counter-rotating vortex pair at its tips which gets lifted downstream guided by the geometry of the dermal denticle. The vortex pairs of neighboring dermal denticles interact with each other such that a pattern of vortices rotating in alternating directions emerges. Regions of alternate vorticity form which undulate back and forth guided by the sawtooth pattern of the trailing edges of the sequence of dermal denticles in flow direction. Undulation occurs in both directions across the main flow direction. The sawtooth pattern formed by the geometry of the dermal denticles is perhaps best visible in the side view, Figure 8d. It shows the flow in the (xz)-plane at the midplane through the dermal denticles. As expected, the recirculating flow is strongest in the midplane of the dermal denticle and becomes weaker in the planes towards the edges of the denticle (data not shown). At a distance of 50 μm the recirculating flow is weak to marginal (data not shown), and at a distance of 100 μm no evidence for flow recirculation is found. The simulated vorticity in the shear layer on top of the dermal denticle is consistent with the μ-PIV measurement. The velocity components in cross-sectional planes are small compared with the streamwise velocity. Despite of their small velocities, the undulating vortices of alternating vorticity appear to be rather stable. This is explained by the vorticity sources at the trailing edges of the dermal denticles. In all, we find that the measurements and simulations are consistent in the overall flow pattern including the coherent vortices and recirculation bubbles.

## 4. Discussion

In Section 2, microscopy images from 31 positions on samples from each of the three shark species were analyzed for areal density and size distribution. Dermal denticles near gills and on the fins were smallest and most densely distributed, whereas the largest dermal denticles were found on the back side of the sharks. One might have suspected a power law dependence of density to dimension, but our data do not show such behavior in a convincing manner.

In Section 3, we selected a few representative examples from the many samples to demonstrate that micro-PIV measurements very near shark skin surfaces are possible and, with the experimental assay discussed here, the micro-PIV measurements provide a close-up view of flow above or around actual dermal denticles. Our study reveals the flow patterns near real shark skin, rather than artificial mock-ups, and we find coherent structures such as the recirculation bubble and coherent eddies convected with the flow. Similar patterns were found in a numerical model, although at much higher Reynolds number. We acknowledge, though, that the microfluidic chambers constructed here do not provide a completely realistic view on the flow patterns near the dermal denticles of a swimming shark, both due to the closeness of nearby surfaces and due to the fact that the skin sample is held flat and rigid.

According to the conjectures in the literature from the 1980s [3], the dermal denticles of the three species that we have investigated should provide defense against ectoparasites or abrasion strength or both in the case of the small-spotted catshark. One study in the literature discusses the possible role of the dermal denticles of this shark species for anti-fouling [19], and demonstrates that structures provide reduced fouling for particular flow speeds. Interestingly, other literature on shark-mimetic structures that show anti-fouling behavior [20,29]—possibly corresponding to the suggested function of defense against ectoparasites—are inspired by the perhaps better-known dermal denticles of fast-moving sharks. Mytilid shells that have also inspired biomimicry for anti-fouling purposes [30] also have ribbed structures, of dimensions similar to the riblets in e.g., dermal denticles of a scalloped hammerhead shark (*Sphyrna lewini*). It is, therefore, tempting to speculate on requirements for dermal denticles to provide anti-fouling function.

The microscopy images in Figure 1 and Figure 3 suggest that the dermal denticles of these two species densely cover the skin of the shark. All dermal denticles also have a few riblets. However, as shown in Figure 2, as well as Figures S3–S5, the dermal denticles of the Greenland shark have a pointed structure with riblets on the denticle surface, ending at the pointed tip. In addition, the density of dermal denticles is sufficiently low that a very large fraction of the skin of Greenland shark, at the μm-mm scale, is without dermal denticles and thus exposed to parasites, if one assumes that the riblets alone provide the anti-fouling function. At several occasions, large 3–4 cm crustacean ectoparasites have been observed on the skin of Greenland shark from both Greenland and Norway [31], but we found no published literature on the matter. It therefore appears that the large dermal denticles do not necessarily seem to protect the Greenland shark completely from such infections. However, as discussed in detail below, it is possible that the vortex flow generated across the skin protect from infections of smaller bacteria or parasites, and future and more detailed and targeted investigations on this particular effect of Greenland shark dermal denticles are called for.

Our alternative hypothesis suggests that the actual structure of the denticles is not decisive for the ability of the denticle to provide anti-fouling, at least not for small fouling agents: Dermal denticles introduce a flow unsteadiness which leads to larger peak velocities intermittently. During a velocity peak, the wall shear stress also increases. We speculate that small fouling agents may be washed away during such a velocity peak. It just requires that only one or a few peaks in the velocity could wash away a parasite that perhaps manages to stay on the shark for lower speeds. Further work is required to investigate the idea, but we provide here first estimates that agree with our speculation: Fouling agents with characteristic sizes of order μm tend to follow the local flow unless they have a way to attach themselves to the shark. This can be estimated from their Stokes number

(1) $$St=\frac{\tau_{mom}}{\tau_{f}}$$

where $\tau_{mom}$ is the characteristic momentum response timescale and $\tau_{f}$ is the characteristic flow timescale which can be thought of as the eddy turnover time. If St≪1, a free fouling agent will follow the local flow (as e.g., airborne seeds), and if St≫1, the fouling agent will not react to the flow (ballistic flight) [32].

The flow time scale, $\tau_{f}$, can be estimated from the length scale *l* and eddy velocity scale $u^{\prime }$ as $\tau_{f}∼l/u^{\prime }$. For $u^{\prime }∼0.01$ m/s and l∼10 μm, one finds $\tau_{f}∼10^{-3}$ s. To estimate the momentum response time of the fouling agent, we assume it to be spherical. Then Newton’s law with the hydrodynamic force is

(2) $$\rho_{ag}\frac{\pi D_{ag}^{3}}{6}\frac{dv_{rel}}{dt}=\frac{1}{2}C_{D}\rho_{w}\frac{\pi D_{ag}^{2}}{4}v_{rel}^{2}$$

We introduce the characteristic momentum response timescale $\tau_{mom}$ in which the fouling agent reacts to the local flow velocity as $\frac{dv_{rel}}{dt}∼\frac{v_{rel}}{\tau_{mom}}$. Solving for $\tau_{mom}$ gives:

(3) $$\tau_{mom}∼\frac{4}{3}\frac{\rho_{ag}}{\rho_{w}}\frac{D_{ag}}{C_{D}v_{rel}}.$$

Introducing the Reynolds number of the fouling agent $Re_{ag}=\frac{v_{rel}D_{ag}}{nu_{w}}$ we get

(4) $$\tau_{mom}∼\frac{24}{C_{D}Re_{ag}}\frac{\rho_{ag}}{\rho_{w}}\frac{D_{ag}^{2}}{18\nu_{w}}$$

where the first fraction approaches unity for Stokes flow and $\rho_{ag}/\rho_{w}∼1$ for typical microorganisms in water. The momentum response time can now be estimated as

(5) $$\tau_{mom}∼\frac{D_{ag}^{2}}{18\nu_{w}}.$$

The detailed geometrical form of the fouling agent changes the numerical factor 18, but not the basic scaling. For water $\nu_{w}∼10^{-6}$ m${}^{2}$/s. For $D_{ag}∼4$ μm, we find the momentum response timescale to be $\tau_{mom}∼10^{-6}$ s. The Stokes number is

(6) $$St∼\frac{D_{ag}^{2}u^{\prime }}{18\nu_{w}l}.$$

As $St∼10^{-3}$ for these parameters, a fouling agent will react to local eddies and flow unsteadiness introduced by dermal denticles. Future work is called for to test this hypothesis.

In summary, we have presented detailed microscopical investigations of dermal denticles from three slowly swimming shark species for which, according to original hypotheses by Reif [3], the dermal denticles provide defense and possibly abrasion strength. While not addressing the postulated overall fluidic function of the dermal denticles, we investigated selected samples from these shark species through micro-PIV measurements of the flow near the dermal denticles, at low Re. The experimental investigations were supplemented by numerical simulations at high Re. In both cases, recirculation bubbles and vortex structures appear in the flow.

## 5. Experimental Methods

### 5.1. Microscopy

Scanning Electron Microscopy (SEM) was performed with electron microscopes at the student facility Nanoteket at DTU Physics (JEOL JCM-6000). Shark skin samples were cut into small pieces (about 5 mm × 5 mm) and positioned in copper stubs for the electron microscope. On some samples, element analysis was carried out as well, based on Energy Dispersive X-ray Spectroscopy available with the electron microscopy equipment. These results agree with the known composition of dermal denticles.

For the determination of areal density of dermal denticles in spiny dogfish, we visualized an area of 3.98 mm × 2.98 mm in the SEM and counted the number of dermal denticles within this area. In addition, measurements of width and length of selected dermal denticles were performed.

For small-spotted catshark and Greenland sharks, the magnification allowed by an optical microscope (Leica DM 2000 LED) equipped with a CCD camera (Leica ICC50 HD) was sufficient. A pre-defined square was selected and the dermal denticles within this area were counted and measurements of denticle width and length performed. Furthermore, we conducted one microscopy session using an optical stereomicroscope (Leica MZ125) equipped with a digital camera (Canon D5 Mk2).

### 5.2. Micro-PIV and Microfluidic Setup

**Figure 9:**
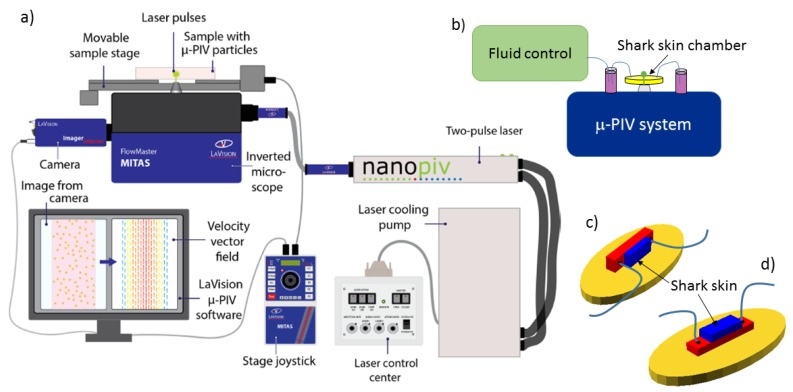
(Color online) Setup, with details. Part (**a**) shows an overview of the elements of the μ-PIV system, consisting of Flowmaster Mitas with a pulsed laser, a sensitive camera, and computer with μ-PIV software. Drawing courtesy of Gitte Frederiksen. Part (**b**) shows a sketch of the combination of fluidics and μ-PIV instrument, with part (**c**) and (**d**) providing details of the microfluidic chambers. The shark skin samples are mounted in the right (**c**) or upper (**d**) rectangular part of the chamber (blue online) with the liquid flowing in the left (**c**) or lower (**d**) slightly larger rectangular part of the chamber (red online). The base (yellow online) illustrates the additional void in the PDMS part that allows for cutting the PDMS before attaching it to a microscope glass slide, then serving as base for the entire chamber and ensuring the closure of the liquid chamber.

Micro-PIV experiments were performed with a commercial Flowmaster Mitas system from LaVision, based on an Nd:YAG double-cavity laser (Litron nano s65—15PIV) and a CCD camera (LaVision Imager Intense). The laser illuminates 0.86 μm or 2 μm diameter fluorescent microspheres (Fluoro-Max R900 and R0200, Thermo Scientific). Imaging was performed with either a 10× or 20× objective (Zeiss, LD Plan-Neofluar, long working distance), in addition to the magnification optics embedded in the Flowmaster Mitas system. Actual dimensions were calibrated using a calibration plate. Image processing and velocity maps were calculated with the software DaVis 8.2. Time-averaged velocity maps were obtained through the method sum of correlation [33], as implemented in DaVis, and recommended as the best averaging method with our experimental conditions. The setup is illustrated in Figure 9a,b.

Microfluidic chambers were based on custom-made 3D printed molds that were cast by soft lithography in polydimethylsiloxane polymer (PDMS; Sylgard 184 Silicone elastomer kit). The PDMS chamber contains a rectangular indentation to create a flow chamber. Within the flow chamber, another indentation is designed to fit a piece of shark skin, 4 mm × 10 mm large, glued to the PDMS with standard two-component epoxy glue. Chambers constructed for control experiments are prepared without the latter indentation. The flow chamber was sealed by a standard microscope slide glued with double sticky tape (ARcare 90106) to the PDMS chamber. Tubing (Teflon tubing, 1.6 mm outer diameter, VWR 228-0738) was mounted in the chamber by drilling holes through the PDMS part prior to gluing the shark skin and to sealing with the microscope slide. PDMS chambers were constructed to allow for 2D micro-PIV measurements both in the plane of the shark skin and with a side view on the dermal denticles. Chamber construction is illustrated in Figure 9c,d.

Microfluidic flow was generated by a pressure-driven pump (Fluigent MFCS-EZ, 4 channel, 345 mbar), and for some experiments, the flow speed was monitored by the Fluigent Flow Rate Platform (Flowboard) with a flowunit size XL.

Control experiments confirmed the overall flow velocity and Poiseuille-flow behavior, with agreement of the numerical value of the average velocity within 50%. These studies also confirmed very low velocities near the channel walls, consistent with a no-slip boundary condition.

### 5.3. Experimental Geometry and Limitations

The flow chamber used for visualization of the flow has dimensions of 4 mm × 18 mm × 1–4 mm, fitting shark skin samples of size 4 mm × 10 mm. To fit the large dermal denticles of the Greenland shark, a chamber depth of 4mm was necessary for those samples. Smaller chamber depths were chosen for the other sharks, i.e., 3 mm for the small-spotted catshark and 1mm for the spiny dogfish. The flow rate platform allows a theoretical limit of maximum 5 mL/min and the pump a maximum applied pressure of 345 mbar.

The natural swimming speeds of the three shark species are not well known. For the Greenland shark, a cruising speed of 0.34 m/s has been suggested [34]. We aimed to perform the experiments at a flow speed that would mimic this estimated cruising speed of a Greenland shark, but were limited by the performance of the pumping system and the requirement for the microfluidic chambers to stay tightly sealed.

## 6. Numerical Methods

Numerical simulations of the flow in the vicinity of dermal denticles aid the understanding of flow phenomena resulting from a geometry with dermal denticles as well as the interpretation of the micro-PIV measurements. Whereas the micro-PIV measurements are two-dimensional, the numerical simulations give access to the flow velocities and vorticities in three dimensions, complementing the measurements. Furthermore, the numerical simulations allow for visualization of the flow patterns in a larger region of space than that investigated microscopically, and with higher Reynolds numbers.

We generated a CAD model simulating an archetypical dermal denticle, modelled to resemble the dermal denticles of the small-spotted catshark. Seven identical dermal denticle models were positioned with a spacing and pattern similar to that observed from SEM microscopy. The areal density of the dermal denticles is 7/mm${}^{2}$. SEM images such as those in Figure S2 were used to build a realistic model of the shape of the dermal denticle including the ribbed structures aligned with the flow on the water-facing side. Details of the geometry of the denticles are provided in Table 1 and Table 2.

**Figure 10:**
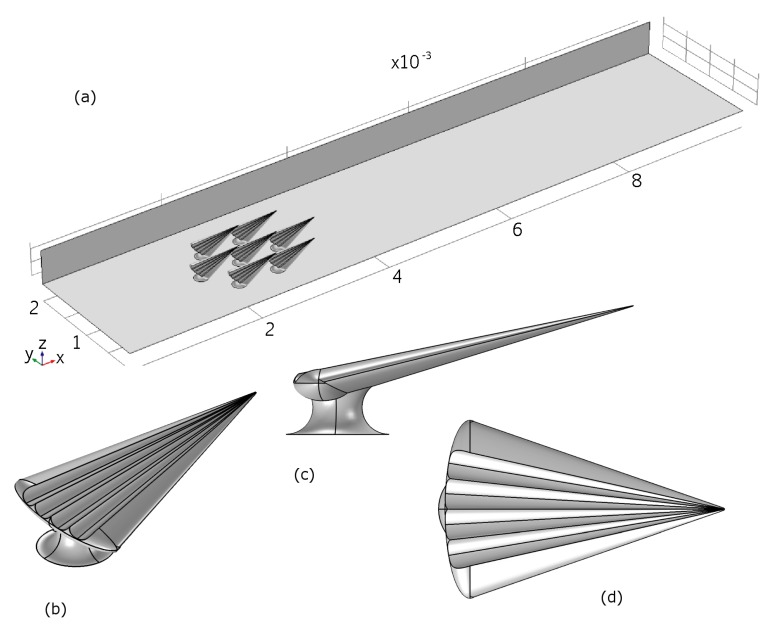
Top: Overview of the modelled geometry in COMSOL. Lower: The CAD model for a single denticle.

The dermal denticles were placed in a channel with the dimensions of the micro-PIV experiment. The simulation model is illustrated in Figure 10, which also illustrates the size of the domain, namely 10 mm along the direction of fluid flow (*x*), 2 mm wide (*y*) and 0.5 mm high (*z*). In the simulations, mirror symmetry in the (x,z)-plane at y=2 mm is assumed, to reduce computational effort, resulting in a final physical domain of 10 mm by 4 mm by 0.5 mm. No-slip boundary conditions are assumed at top ((x,y)-plane at z=0.5mm), bottom ((x,y)-plane at z=0mm) and sides ((x,z)-planes at y=0mm and y=4mm), as well as at the surface of the dermal denticles. The center of the first dermal denticle is positioned 2 mm after the inlet. At the inlet, a fully developed turbulent velocity profile is assumed, and deduced from a simulation of the steady-state solution in a domain of the same cross-sectional area and boundary conditions, but without dermal denticles. At the outlet, flux-conserving zero-gradient conditions on the flow velocities were assumed.

The size of the domain is chosen to fit the experimental conditions, and thus boundary effects that may influence the flow measured would also be found in the numerical model.

The flow is simulated using the incompressible Reynolds-averaged Navier-Stokes equations of Newtonian fluids which are closed using a standard *k*-ϵ-turbulence model. The Reynolds number is Re∼5000 based on typical length scales of dermal denticles and flow velocities, providing a Reynolds number orders of magnitude larger than the limitations imposed in the experiments. The equations were discretized using the finite element method implemented in the commercially available software COMSOL. We used an unstructured grid with tetrahedral cells close to the dermal denticles and a structured grid downstream. The grid size was determined after numerical grid convergence tests. In all, the grid had just over 500.000 cells.

## 7. Biological Samples

For investigations of samples from the various positions on the shark body as schematically illustrated in Figure S1, and described in Table 3, shark skin samples from five sharks were obtained:

- Greenland shark (*Somnosius microcephalus*), female, total body length 470 cm, caught 7 July 2015, during Greenland Institute of Natural Resources annual fish survey from trawler RV Pâmiut.
- Small-spotted catshark (*Scyliorhinus canicula*), male, total body length 72 cm, raised at Marine Biological Laboratory, University of Copenhagen.
- Small-spotted catshark, female, total body length 59 cm, raised at Marine Biological Laboratory, University of Copenhagen. Samples from position *b4* and *c1* only.
- Spiny dogfish (*Squalus acanthias*), female, total body length 76 cm, by-catch and bought at local fishmonger in Copenhagen spring 2016.
- Spiny dogfish, female, total body length 99 cm, by-catch, frozen from Nordsøen Oceanarium, Hirtshals. Unknown date of capture.

For the Greenland shark, samples of varying size (5–15 cm × 5–15 cm) were cut immediately upon capture and subsequently frozen. For the other skin samples, whole sharks were frozen until partially thawed to allow cutting samples of varying size (3–15 cm × 3–15 cm) from the sharks. These samples were refrozen and kept at −18 ${}^{\circ }$C until use. For subsequent microscopy, or mounting in microfluidic chambers, smaller samples were cut as described in Section 2. Naturally, samples thawed during handling in the microscopes or in the microfluidic chambers. They were investigated as fast as possible and kept in the refrigerator for at maximum a few weeks.

## Figures and Tables

**Table 1:** Summary of the geometric values for the dermal denticles in our experimental investigations of a catshark sample, and in the numerical model. In the experiments, the height was not found, but the numerical model used an estimation based on the available SEM images.

	Density [no/mm${}^{2}$]	Length [μm]	Width [μm]	Height [μm]
SEM images	7.3 ± 0.5	685 ± 139	394 ± 73	
Model	7	600	420	100

**Table 2:** Details of the riblets in the experimental investigations of the catshark dermal denticles, and the values chosen in the numerical model. The quantity *h* is riblet height, and *s* is distance between riblets. According to literature, the ratio h/s is indicative for the circulation patterns in the flow [6].

	h [μm]	s [μm]	h/s
SEM images	27 ± 2	88 ± 15	0.31 ± 0.08
Model	25	80	0.31

**Table 3:** Specification of sample positions from the sharks.

n1	Nose	pj	Pectoral fin joins to body
b1 b2 b3	Anterior body: between mouth and gills	b4, b5 b6, b7 b7, b8	Mid body: inter-dorsal range
b10 b11 b12	Posterior body: between dorsal fin and caudal fin	d1 d2 d3	Dorsal fin
g1	Before gills	g2	After gills
p1 p2 p3	Topside of pectoral fin	p4 p5 p6	Underside of pectoral fin
c1 c2 c3	Upper caudal fin	c4 c5 c6	Lower caudal fin

## Supplementary Materials

The following are available online at https://www.mdpi.com/2313-7673/4/2/38/s1, Figure S1: Map of positions on a generic shark, Figures S2–S5: selected examples of SEM and optical microscopy images of dermal denticles from the small-spotted catshark and the Greenland shark. Figure S6: Plot of areal density (Greenland shark and small-spotted catshark), Figure S7: Plot of mean length and width of dermal denticles of a spiny dogfish. Figure S8: μ PIV recording near a dermal denticle from the small-spotted catshark. Explanatory text.

## Abbreviations

The following abbreviations are used in this manuscript:

MDPI	Multidisciplinary Digital Publishing Institute
DOAJ	Directory of open access journals
PDMS	PolyDiMethylSiloxane polymer
PIV	Particle Image Velocimetry
SEM	Scanning Electron Microscopy

## References

[1] Hamlett W.C. (1999). Sharks, Skates and Rays: The Biology of Elasmobranch Fishes.

[2] Castro J.I., Peebles D. (2011). The Sharks of North America.

[3] Reif W.E. (1985). Squamation and Ecology of Sharks. Cour. Forschungsinstitut Senckenberg.

[4] Raschi W., Tabit C. (1992). Functional aspects of Placoid Scales: A review and update. Aust. J. Mar. Freshw. Res..

[5] Bechert D.W., Bruse M., Hage W., Meyer R. (2000). Fluid mechanics of biological surfaces and their technological application. Die Naturwissenschaften.

[6] Dean B., Bhushan B. (2010). Shark-skin surfaces for fluid-drag reduction in turbulent flow: A review. Philos. Trans. Ser. A Math. Phys. Eng. Sci..

[7] Oeffner J., Lauder G.V. (2012). The hydrodynamic function of shark skin and two biomimetic applications. J. Exp. Biol..

[8] Wen L., Weaver J.C., Lauder G.V. (2014). Biomimetic shark skin: Design, fabrication and hydrodynamic function. J. Exp. Biol..

[9] Lang A.W., Jones E.M., Afroz F. (2017). Separation control over a grooved surface inspired by dolphin skin. Bioinspir. Biomim..

[10] Lauder G.V., Wainwright D.K., Domel A.G., Weaver J.C., Wen L., Bertoldi K. (2016). Structure, biomimetics, and fluid dynamics of fish skin surfaces. Phys. Rev. Fluids.

[11] Lang A.W., Motta P., Hidalgo P., Westcott M. (2008). Bristled shark skin: A microgeometry for boundary layer control?. Bioinspir. Biomim..

[12] Lang A., Motta P., Habegger M.L., Hueter R., Afroz F. (2011). Shark Skin Separation Control Mechanisms. Mar. Technol. Soc. J..

[13] Motta P., Habegger M.L., Lang A., Hueter R., Davis J. (2012). Scale morphology and flexibility in the shortfin mako Isurus oxyrinchus and the blacktip shark Carcharhinus limbatus. J. Morphol..

[14] Clos K.T.D., Lang A., Devey S., Motta P.J., Habegger M.L., Gemmell B.J. (2018). Passive bristling of mako shark scales in reversing flows. J. R. Soc. Interface.

[15] Wen L., Weaver J.C., Thornycroft P.J.M., Lauder G.V. (2015). Hydrodynamic function of biomimetic shark skin: Effect of denticle pattern and spacing. Bioinspir. Biomim..

[16] Domel A.G., Saadat M., Weaver J.C., Haj-Hariri H., Bertoldi K., Lauder G.V. (2018). Shark skin-inspired designs that improve aerodynamic performance. J. R. Soc. Interface.

[17] Reif W.E. (1982). Morphogenesis and function of the squamation in sharks. Neues Jahrbuch für Geologie und Paläontologie.

[18] Fletcher T., Altringham J., Peakall J., Wignall P., Dorrell R. (2014). Hydrodynamics of fossil fishes. Proc. R. Soc. B Biol. Sci..

[19] Sullivan T., Regan F. (2011). The characterization, replication and testing of dermal denticles of Scyliorhinus canicula for physical mechanisms of biofouling prevention. Bioinspir. Biomim..

[20] Schumacher J.F., Carman M.L., Estes T.G., Feinberg A.W., Wilson L.H., Callow M.E., Callow J.A., Finlay J.A., Brennan A.B. (2007). Engineered antifouling microtopographies—Effect of feature size, geometry, and roughness on settlement of zoospores of the green alga Ulva. Biofouling.

[21] Bers A.V., Wahl M. (2004). The influence of natural surface microtopographies on fouling. Biofouling.

[22] Magin C.M., Cooper S.P., Brennan A.B. (2010). Non-toxic antifouling strategies. Mater. Today.

[23] Kirschner C.M., Brennan A.B. (2012). Bio-Inspired Antifouling Strategies. Annu. Rev. Mater. Res..

[24] Lang A.W., Bradshaw M.T., Smith J.A., Wheelus J.N., Motta P.J., Habegger M.L., Hueter R.E. (2014). Movable shark scales act as a passive dynamic micro-roughness to control flow separation. Bioinspir. Biomim..

[25] Afroz F., Lang A., Habegger M.L., Motta P., Hueter R. (2016). Experimental study of laminar and turbulent boundary layer separation control of shark skin. Bioinspir. Biomim..

[26] Adrian R.J. (1991). Particle imaging techniques for experimental fluid mechanics. Annal. Rev. Fluid Mech..

[27] Santiago J.G., Wereley S.T., Meinhart C.D., Beebe D.J., Adrian R.J. (1998). A particle image velocimetry system for microfluidics. Exp. Fluids.

[28] Cierpka C., Kähler C.J. (2012). Particle imaging techniques for volumetric three-component (3D3C) velocity measurements in microfluidics. J. Vis..

[29] Carman M.L., Estes T.G., Feinberg A.W., Schumacher J.F., Wilkerson W., Wilson L.H., Callow M.E., Callow J.A., Brennan A.B. (2006). Engineered antifouling microtopographies - Correlating wettability with cell attachment. Biofouling.

[30] Bers A.V., Diaz E.R., da Gama B.A.P., Vieira-Silva F., Dobretsov S., Valdivia N., Thiel M., Scardino A.J., McQuaid C.D., Sudgen H.E., Thomason J.C., Wahl M. (2010). Relevance of mytilid shell microtopographies for fouling defence—A global comparison. Biofouling.

[31] Nielsen J. (2018). Personal communication.

[32] Salewski M., Fuchs L. (2007). Consistency issues of Lagrangian particle tracking applied to a spray jet in crossflow. Int. J. Multiph. Flow.

[33] Meinhart C.D., Wereley S.T., Santiago J.G. (2000). A PIV Algorithm for Estimating Time-Averaged Velocity Fields. J. Fluids Eng..

[34] Watanabe Y.Y., Lydersen C., Fisk A.T., Kovacs K.M. (2012). The slowest fish: Swim speed and tail-beat frequency of Greenland sharks. J. Exp. Mar. Biol. Ecol..

