# Supplementary material for: Dermal Denticles of Three Slowly Swimming Shark Species: Microscopy and Flow Visualization
Source: Biomimetics (Basel). 2019 May 24;4(2):38. doi: 10.3390/biomimetics4020038 (PMC6631580; doi:10.3390/biomimetics4020038)
Supplement: Supplementary file 1 [file biomimetics-04-00038-s001.pdf]

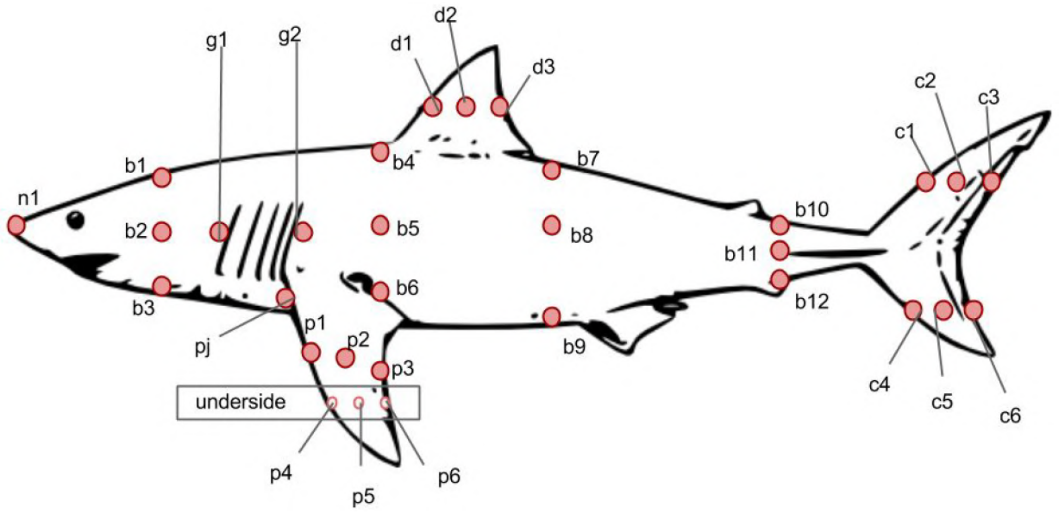

Figure S1. Map of skin sample positions on a generic shark.

.

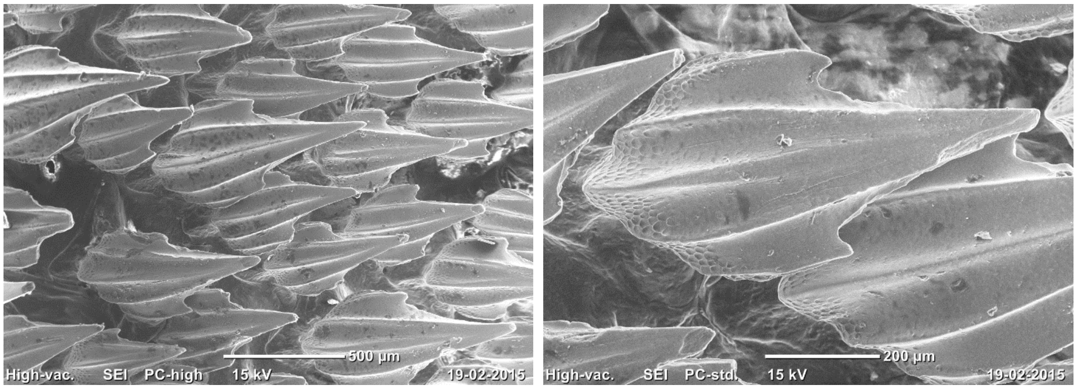

Figure S2. SEM images of dermal denticles from area *c1* of a small spotted catshark (female, TL 59 cm). Scalebars are 500 μm (left image) and 200 μm (right image). Images courtesy of Thomas Erik Bohn Smitshuysen and Emil Christian Jensen.

Supplementary information

Fig. S1 shows the detailed map of skin sample positions, on a generic shark. Figs. S2-S3 show detailed views of dermal denticles obtained by SEM. Figs. S4-S5 show images of dermal denticles from the greenland shark, obtained by optical microscopy.

For the Greenland shark, the areal density is found by counting the number of denticles in a 15mm x 15mm area, visualized by optical microscopy. For the sections with multiple lengths measured, the mean of the lengths are calculated. These results are shown in Fig. S6(a). Even though the points do not form a perfect line they appear in a clear tendency of large denticles associated with low density.

For the small spotted catshark, detailed images of the dermal denticles were obtained by SEM. From the microscope images of the denticles, the length of a few denticles and the total number of denticles in each frame was noted. Fig. S6(b) shows

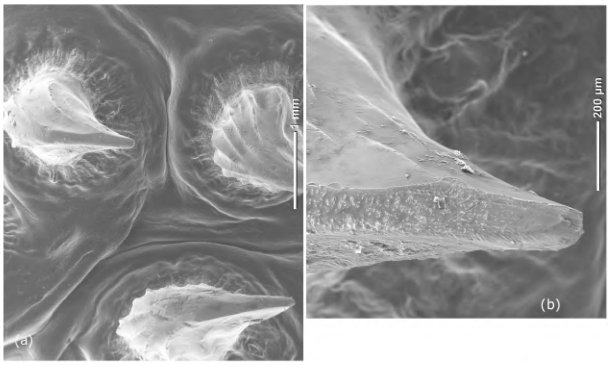

Figure S3. SEM pictures of a denticle from the Greenland shark (female, TL 470cm). This denticle was positioned right in front of the pectoral fin, position *pj*. In the close-up in (b) it appears that the denticle has a smooth and a rough side. Scalebars are 1mm in (a) and 200μm in (b). The shape of the dermal denticle is quite typical for all areas (other than the nose) investigated.

the areal density versus the length of denticles in a semi-logarithmic coordinate system. As one could expect, the longer the denticles, the lower their areal density, yet we cannot conclude a specific power law dependence.

The dermal denticles of the spiny dogfish were also investigated by SEM. Results are collected to extract the areal density as displayed in Fig. S7.

We searched for signs of bristling in the small-spotted catshark during a single microscopy session, conducted on an alive, adult shark of a length of about 70 cm, generously made available to us by The Øresund Aquarium. The shark was positioned under a stereomicroscope in dry conditions for a brief amount of time, during which we visualized a region on the tail and a region of the dorsal fin. Bending the fin was attempted, in order to possibly induce a condition that would force the dermal denticles to bristle should they have a natural tendency to do so. No sign of bristling was observed, however.

One μ-PIV recording is shown in Fig. S8. As explained in the main text, the flow was kept at 5ml/min throughout the whole experiment through the chamber with a cross-section of 3mm $\times$ 4mm, corresponding to a flow velocity of approximately 7 mm/s along the chamber. With a typical length of 600 μm of the dermal denticle as the length scale, this flow velocity corresponds to a Reynolds number of 4. Fluorescent seeding particles with a diameter of 0.86 μm were added to the water used in the flow experiment. Measurements where conducted with the 20$\times$ objective. In all of these, 30 picture pairs were taken with a time delay of 8 ms, 10 ms and 12 ms between the paired images. A vector field is calculated for each of the 30 picture pairs using standard PIV processing tools, thus creating a short movie of 30 frames. The relatively low number of picture pairs was a compromise that ensured constancy in the overall imposed flow while still obtaining trustworthy micro-PIV vector fields. A time averaged vector field of the 30 frames was then calculated. At this relatively low flow speed, the streamlines point almost straight away from the skin but vortices appear under the tips of the denticles

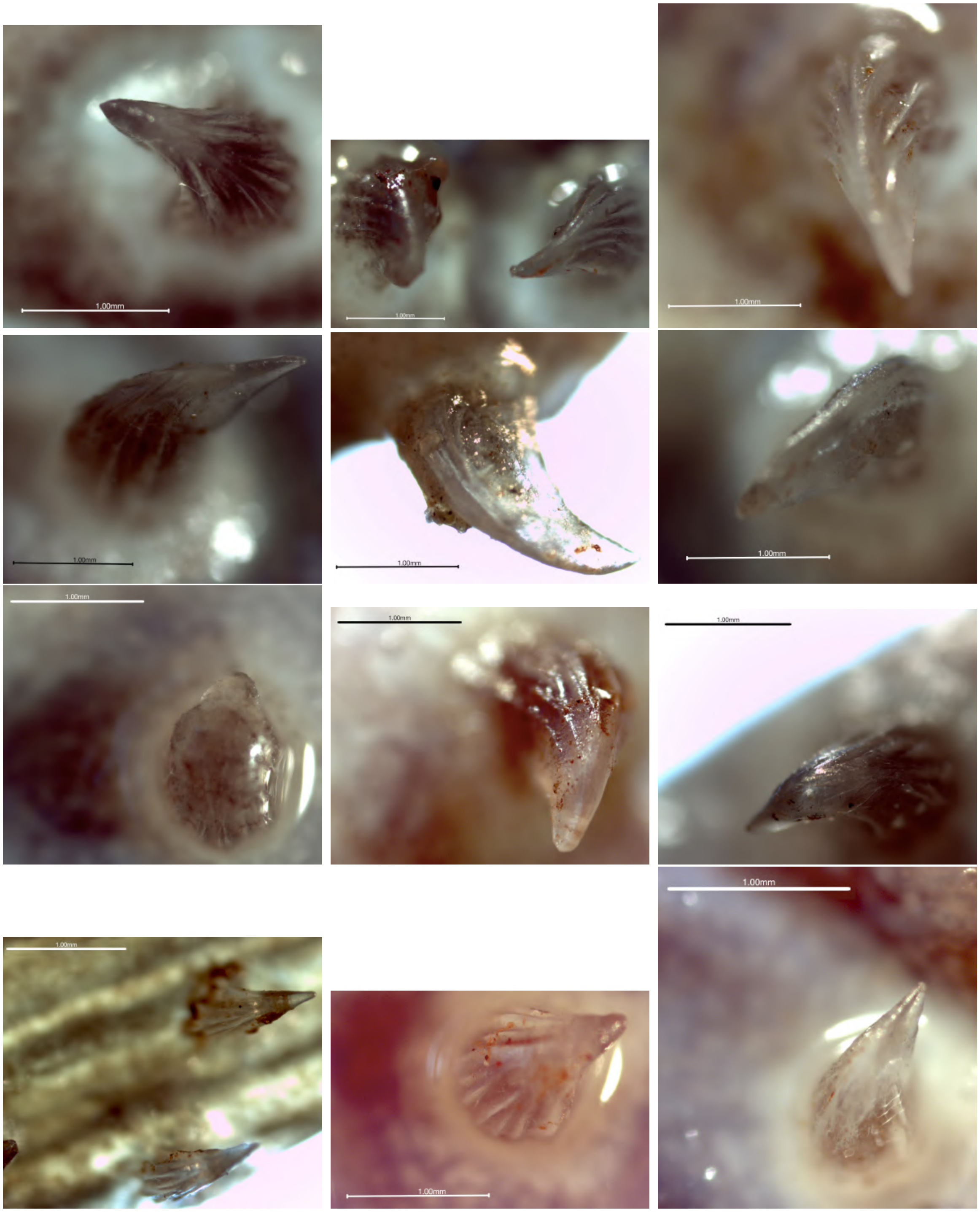

Figure S4. Examples of optical microscopy images of dermal denticles from a Greenland shark (female, TL 470 cm), most images have only one dermal denticle within the field of view. Scale bar is 1mm in all cases. Upper line: Dermal denticles from area *b1*, *b2*, and *b4*. Second line: Dermal denticles from area *b5*, *b6*, and *b9*. Third line: Dermal denticles from area *b10*, *b11*, and *b12*. Fourth line: Dermal denticles from area *c3*, *c4*, and *c5*.

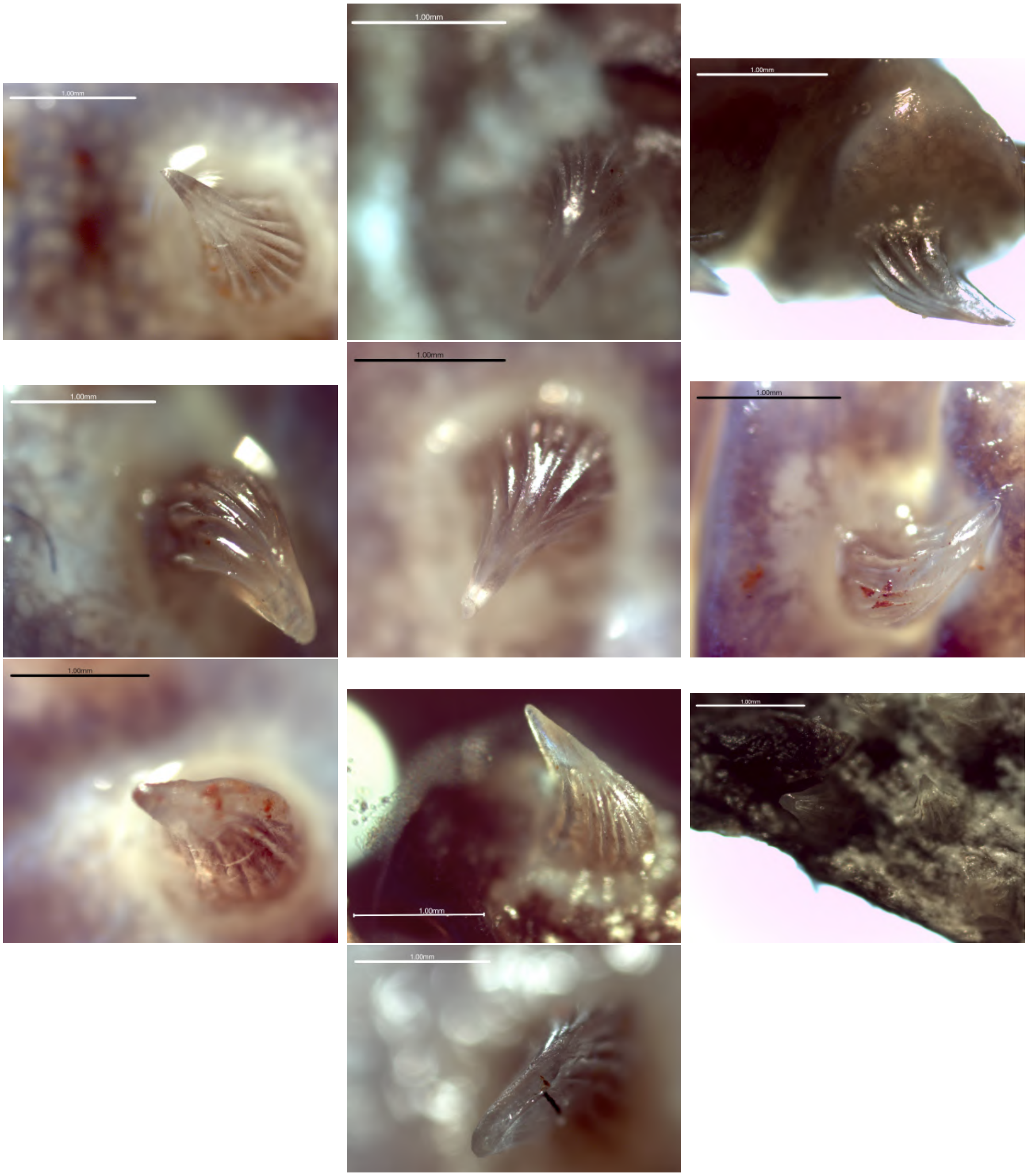

Figure S5. Examples of optical microscopy images of dermal denticles from a Greenland shark (female, TL 470 cm), most images have only one dermal denticle within the field of view. Scale bar is 1mm in all cases. Upper line: Dermal denticles from area *d1*, *d2*, and *d3*. Second line: Dermal denticles from area *g2*, *p1*, and *p3*. Third line: Dermal denticles from area *p4*, *p5*, and *p6*. Fourth line: Dermal denticle from area *pj*.

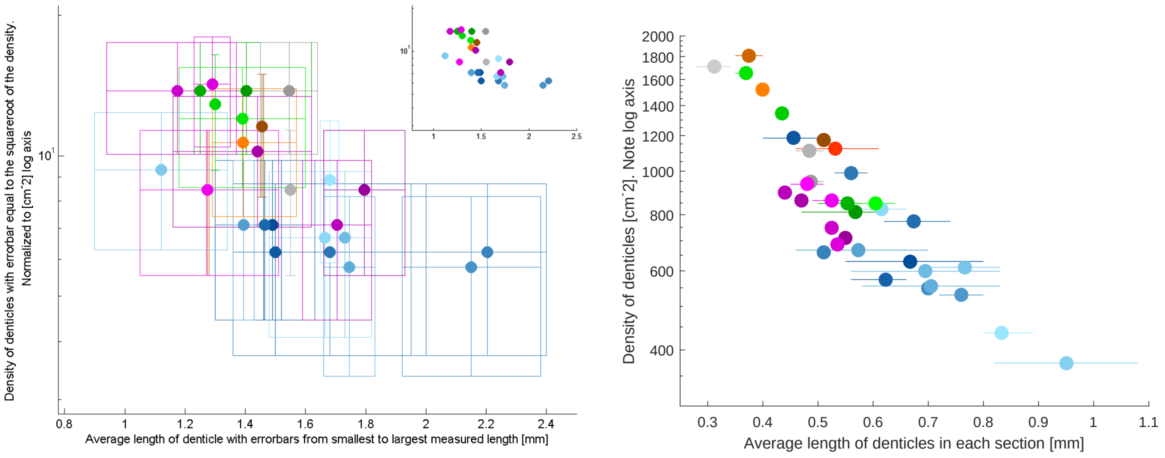

Figure S6. Plot of areal density of denticles around the body of different sharks against their average length in the measurement area. Part (a) gives results for a greenland shark (female, TL 470 cm). Notice that the y-axis is logarithmic. The errorbars for the length goes from the smallest measured to the highest measured length in the specific section. When assigning errorbars for the density itself, we assume that counting statistics is Poissonian, i.e., the error in the count number is assigned to be equal to the square-root of the count. In the main plot, errorbars are shown as a box connected to the corresponding point, insert shows the same data without the error-box. The colours represent different areas of the shark; blue is the body, green is the caudal fin, orange is the dorsal fin, grey is by the gills and purple is the pectoral fin. Darker colour signify a higher number in the area name e.g. *b12* is pictured in darker blue than *b4*. Note that *pj* is in a lighter purple than *p1*. Part b) shows results for denticles of a small spotted catshark (male, TL 72 cm), with a similar color coding. Horizontal error bars go from smallest measured to longest measured length. Vertical error bars are too small to see in the plot.

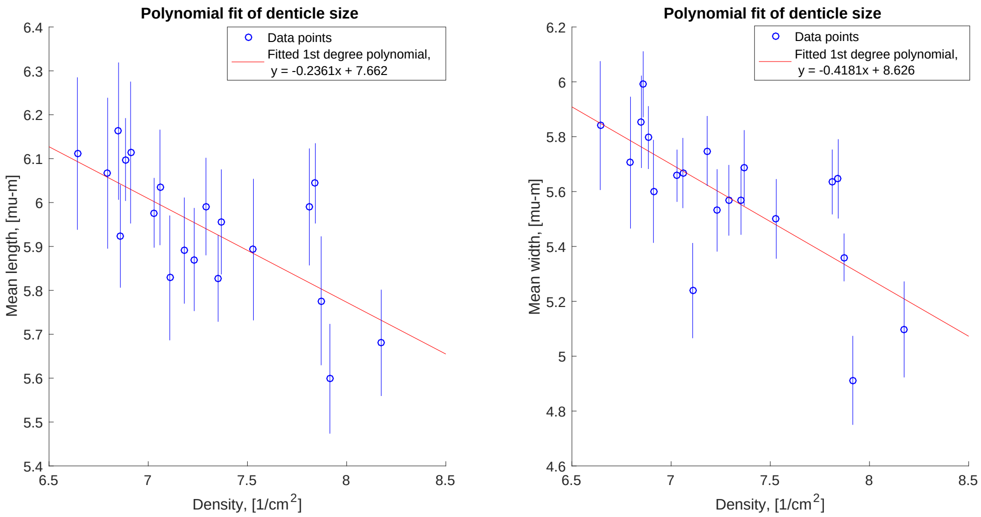

Figure S7. The mean length and mean width of the dermal denticles of the spiny dogfish (female, TL 76 cm) plotted versus the density. The relation is fitted with a 1st degree polynomial. Both axes are on logarithmic scale.

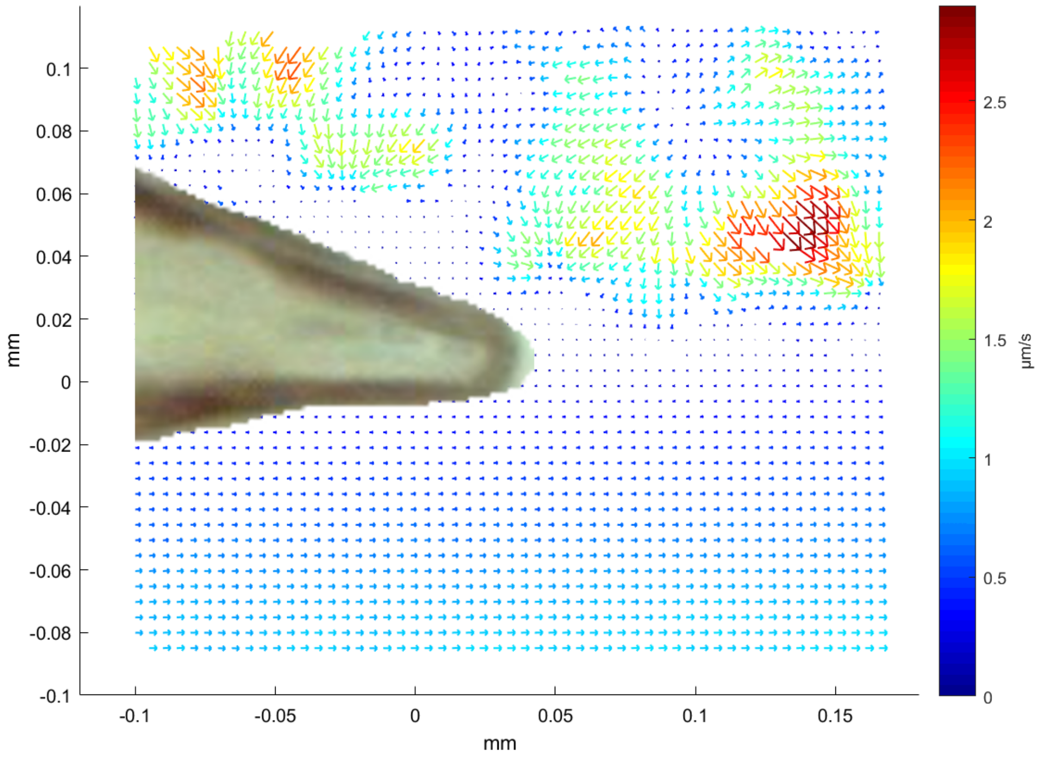

Figure S8. Vector field of flow near denticles from the small spotted catshark (male, TL 72 cm, denticle from pectoral fin) with a 20× objective. A reference vector of speed 0.01m/s is shown in top corner. Denticles are shown upside down and pointing to the right, a microscope image of a denticle from the same area, scaled to match the dimensions of the field of view, is overlaid to roughly illustrate the position of the denticle which is otherwise hardly visible in the image. The water is flowing to the right and is illustrated by vector arrows in colours between blue (slow) and red (fast), ranging between 0 μm/s and 3 μm/s, as indicated in the color map. The field of view covers an area of width 285 μm and height 215 μm.
